# Ex Vivo, In Vitro and In Vivo Bone Health Properties of Grana Padano Cheese

**DOI:** 10.3390/foods14020273

**Published:** 2025-01-16

**Authors:** Cristina Martelli, Luisa Ottobrini, Anita Ferraretto, Paola Bendinelli, Stefano Cattaneo, Fabio Masotti, Milda Stuknytė, Margherita Dall’Asta, Angelo Del Sole, Ivano De Noni, Filippo Rossi

**Affiliations:** 1 Department of Pathophysiology and Transplantation, University of Milan, 20054 Milan, Italy; cristina.martelli@unimi.it (C.M.); luisa.ottobrini@unimi.it (L.O.); 2 Bioimaging and Complex Biological Systems (IBSBC), National Research Council (CNR), 20054 Milan, Italy; 3 Department of Biomedical Sciences for Health, University of Milan, 20133 Milan, Italy; anita.ferraretto@unimi.it (A.F.); paola.bendinelli@unimi.it (P.B.); 4 Department of Food, Environmental and Nutritional Sciences, University of Milan, 20133 Milan, Italy; stefano.cattaneo@unimi.it (S.C.); fabio.masotti@unimi.it (F.M.); 5 Unitech COSPECT—COmprehensive Substances characterization via advanced sPECTtroscopy, University Technological Platform, University of Milan, 20133 Milan, Italy; milda.stuknyte@unimi.it; 6 Department of Animal Science, Food and Nutrition, Università Cattolica del Sacro Cuore, 29122 Piacenza, Italy; margherita.dallasta@unicatt.it (M.D.); filippo.rossi@unicatt.it (F.R.); 7 Department of Health Sciences, University of Milan, 20146 Milan, Italy; angelo.delsole@unimi.it

**Keywords:** calcium, caseinophosphopeptides, Ussing chambers, Caco-2/HT-29 cells, SaOS-2 cells, CD1 mice, bone remodeling

## Abstract

Grana Padano (GP) is an Italian hard cooked cheese characterized by a long ripening process and high protein and Ca contents. After in vitro static simulated gastrointestinal digestion, GP digest contained caseinophosphopeptides that were 6 to 24 amino acids in length, including tri-phosphorylated species incorporating the pSer-pSer-pSer-Glu-Glu cluster. Using rat ileum tissue, the digest was used to assess Ca absorption ex vivo, which showed significantly better results for the GP digest in comparison to the CaCO_3_ aqueous solution. An in vitro intestinal model based on Caco-2/HT-29 cell co-culture was able to mimic Ca absorption from GP digest, with Ca-rich water as a control. The metabolite-containing medium was then used to treat osteoblast-like SaOS-2 cells. As a consequence, metabolized GP digest significantly increased the number of osteoblasts, whereas the metabolized water did not exert this effect. Finally, the mice were fed diets containing GP or CaCO_3_ and pea isolate and the in vivo outcomes were assessed through fluorescent probe and computed tomography. Mice fed a diet containing GP showed a higher increase in bone remodeling and volume in comparison to those fed a control diet containing CaCO_3_ and pea isolate. Overall, the ex vivo, in vitro and in vivo experiments highlighted the effectiveness of GP in improving Ca absorption, osteoblast proliferation and bone remodeling and volume.

## 1. Introduction

Dairy products are generally recognized as dietary sources that provide bone-beneficial nutrients, such as calcium (Ca), proteins and mineral-carrier peptides [1]. These nutrients have a paramount role in healthy bone morphology [2], and their concentrations are higher in cheese than in other dairy products. Grana Padano (GP) is an Italian hard cooked cheese made from raw bovine milk. It is characterized by a long ripening process and high protein and Ca contents (about 32.0 and 1.2 g/100 g, respectively) [3]. The manufacturing and ripening processes influence the GP digestibility and hence the bioavailability of nutrients. For instance, extensive research has shown that GP represents a source of caseinophosphopeptides (CPP), which are released from bovine casein (CN) upon the action of proteases and peptidases originating from milk, milk-clotting enzymes, cheese microorganisms and the human digestive tract [4,5,6]. Indeed, CN is characterized by a primary structure in which (repeated) residues of phosphoserine (SerP) are present [7]. These residues mainly occur as a SerP-SerP-SerP-Glu-Glu (sssEE) motif. This is a highly negatively charged “acid domain” that remains intact in CPP and acts as a mineral carrier by linking bivalent ions both in vitro and in vivo [8]. Consequently, very soluble metal organophosphates are formed, thus preventing Ca insolubilization [9] and enhancing its absorption by intestinal cells. Moreover, CN raises the serum level of insulin-like growth factor 1 (IGF-1), which in turn increases osteoblast activity and induces the renal re-absorption of phosphates [10,11,12,13].

An adequate Ca intake is strongly recommended for bone health, regardless of dietary habits [2]. Nonetheless, the role in bone health that the Ca present in different food matrices is debated, especially in relation to the effects on Ca absorption and bone mineralization [14,15]. Despite this, cheese remains a main source of dietary Ca, and its consumption may guarantee an adequate intake of this mineral, which also plays a role in blood clotting and muscle contraction, as well as heart and nerve functions [16,17]. As a matter of fact, only 1% of the human body’s Ca is stored in districts other than bone tissue; the latter consists of 50% proteins and 50% Ca phosphate [18]. Along with genetic traits and physical activity, an adequate ingestion of this mineral is considered to be a major determinant for reaching peak bone mass [19]. The daily Ca requirement increases during different stages of life, ranging from 1000 to 1200 mg depending on age, sex, pregnancy or lactation [20]. Most Western populations have an average Ca intake markedly below the recommended dietary daily allowance, even if the relation between low Ca ingestion and age-related bone loss has been clearly stated [21]. In this situation, the body draws Ca from bones through osteoclast action, and an unbalanced osteoblast/osteoclast activity occurs [22]. To date, osteoporosis affects about 200 million people worldwide [23].

Despite cheeses being recognized as sources of nutrients, especially Ca, which has a paramount role in bone health, their consumption is sometimes discouraged because of the perception of excess calories and fat intake. Consequently, in recent decades, Ca-rich drinking water has been proposed as a calorie-free Ca-source alternative to dairy products in order to satisfy the daily requirement for this mineral [24].

However, not enough studies have compared cheese and water addressing Ca absorption and bone remodeling effects. In the present work, we submitted the GP cheese to in vitro static simulated gastrointestinal digestion (SGID) and evaluated the CPP profile of the obtained digest. Subsequently, we used the digest to assess Ca absorption ex vivo, and the effect on osteoblast proliferation in vitro. As controls, an aqueous solution of CaCO_3_ and commercial Ca-rich drinking water were used during the ex vivo and in vitro experiments. Finally, we implemented GP to formulate diets for feeding mice with the aim to evaluate the bone density and remodeling in vivo when GP or pea isolate and CaCO_3_ were used as the protein or Ca source.

## 2. Materials and Methods

### 2.1. Grana Padano Cheese Sample, CaCO_3_ Aqueous Solution and Ca-Rich Drinking Water

A sample of GP cheese matured for 10 months was used in the present study. It was kindly provided by the “Consorzio Tutela Grana Padano” (Desenzano del Garda, Italy). This sample was representative of the most common type of GP sold in the Italian market. The Ca, water and protein contents (g/100 g) of the GP sample were 1.18, 34.0 and 32.2, respectively. The calcium content was determined by inductively coupled plasma optical emission spectroscopy (ICP-OES), according to the method EPA 6010d [25]. The total solids were determined by drying at 102 °C [26], and total nitrogen by the Kjeldahl method [27].

An aqueous (Milli-Q-treated water) solution of CaCO_3_ (1.35 mM) (Merck, Darmstadt, Germany) and a commercial Ca-rich drinking water (11 mM Ca, as declared on the label) were used as controls for the evaluation of Ca bioavailability during ex vivo and in vitro experiments, respectively.

### 2.2. In Vitro Static Simulated Gastrointestinal Digestion

The in vitro SGID of the GP sample was performed according to the INFOGEST protocol [28]. Simulated salivary (SSF), gastric (SGF) and duodenal (SDF) fluids were prepared accordingly. In detail, the GP sample (5 g) was grinded in the presence of 5 mL of SSF at pH 7.0 for 2 min to reproduce chewing. The derived bolus was supplemented with 10 mL of SGF-containing porcine pepsin (1000 U/mL of SGF) and rabbit gastric lipase (30 U/mL of SGF). The gastric digestion step was performed at 37 °C for 2 h at pH 3.0 (adjusted with 1 N HCl). Subsequently, 20 mL of SDF and bile salts (10 mM) were added to the digest. The enzymes used for intestinal digestion were porcine trypsin (200 U/mL SDF), bovine chymotrypsin (50 U/mL SDF), porcine intestinal lipase (1000 U/mL SDF) and co-lipase (lipase/colipase at 1:2 molar ratio). The intestinal phase was conducted at 37 °C for 2 h at pH 7.0 (1 N NaOH), and it was blocked by the addition of the protease inhibitor AESFB (Roche, Mannheim, Germany). The cheese sample was subjected to three replicate digestions on the same day. Control digestions without cheese were also performed. The digests were immediately frozen, then lyophilized and stored at −40 °C. The enzymes, chemicals and bile salts were from Merck.

### 2.3. Characterization of the Qualitative CPP Profile of GP Cheese Digest

The CPP were extracted from cheese digest by selective precipitation with CaCl_2_ (Merck) and ethanol (Merck) according to [29]. Briefly, freeze-dried cheese digest was resuspended in Milli-Q-treated water, and CPP were precipitated by adding 10% (*w*/*v*) CaCl_2_ solution (20 mol/mol protein) and, subsequently, an equal volume of 99% (*v*/*v*) ethanol. After washing (50%, *v*/*v* ethanol) and centrifugation, the dried (under N_2_ flow) pellet was resuspended in ultrapure water containing 0.1% formic acid (FA), filtered through a 0.2 μm pore size PVDF syringe filter membrane (Merck) and stored at −40 °C before analysis by ultra-performance liquid chromatography/high-resolution mass spectrometry (UPLC/HR-MS).

Extracts of CPP from cheese digest were separated by reverse phase UPLC, and peptides were identified by HR-MS, as already described [30]. Briefly, an Acquity UPLC module (Waters, Milford, MA, USA) was coupled through a HESI probe to a Q Exactive Orbitrap mass spectrometer (Thermo Fisher Scientific, San Jose, CA, USA). The extracts were separated on an Acquity UPLC BEH300 C18 column (150 × 2.1 mm, 1.7 μm) (Waters), and the peptides were eluted upon linear elution gradient (from 5% to 60% of acetonitrile containing 0.1% FA in 36 min). The LC eluate was analyzed by HR-MS in data-dependent tandem MS (MS/MS) mode. The data were processed and CPP were identified using Proteome Discoverer software (v1.4, Thermo Fisher Scientific). Full MS and data-dependent MS/MS spectra were processed using the *Bos taurus* database (UniProt taxon ID 9913), considering CN and its genetic variants [7].

### 2.4. Ex Vivo Evaluation of Ca Absorption

The intestinal absorption of Ca was tested ex vivo using Ussing chambers made up of a thermostatic structure with 6 cells and equipped with an oxygen infusion system. Segments of rat ileum tissue were fixed in half-cells within 1 h of collection. The rat ileum segments were from adult animals as a donation of non-used organs from the animal facility of the Department of Pharmacological and Biomolecular Sciences of the University of Milan. After sampling, the tissue was placed in a Krebs buffer (Merck) maintained on ice and placed onto an Ussing chamber within 60 min from collection from the animal body. Each cell was made up of two half-cells that represented the two sides of the intestinal mucosa: the mucosal one inside the rat ileum and the serosal tissues, i.e., the side not in contact with the intestinal lumen. To adequately supply the intestinal cells with nutrients, keeping them alive and vital, a saline medium was placed in both cells [31].

The two half-cells were then joined with a steel O-ring. The chamber of each half-cell was filled with saline medium and oxygenated by infusion with a gaseous mixture (95/5, O_2_/CO_2_). Calcium absorption was determined using in vitro digested GP and an aqueous solution of CaCO_3_ (1.35 mM). A total of 5 mL of saline medium was placed in each Ussing chamber. Subsequently, 1 mL of CaCO_3_ solution or 12 µL of GP digest were added to each chamber (mucosal side). The Ca content of each mucosal chamber was 468 µg for the experiments with the CaCO_3_ solution and 498 µg for those containing digested GP. The tissues were incubated for 1.5 h. Finally, the Ca contents of both chambers were determined by the approved method [25]. Calcium absorption (Ca_abs_ %) was determined using the following equation:

$$Caabs\%=\frac{Ca\;serosal\;half-chamber}{Ca\;mucosal\;half-cell+Ca\;serosal\;half-chamber}$$

Using this equation, the fractional (%) absorption of Ca was assessed, i.e., the amount of mineral contained in the mucosal side calculated by considering the Ca originally present in the saline medium placed in the mucosal side and serosal cells [32]. For each Ca source (digested GP or CaCO_3_ solution), 9 replicates were obtained, 3 for each of the 3 d incubation.

### 2.5. In Vitro Effects of GP Digest on Viability and Proliferation of Osteoblast-like Cells

Caco-2 (BS TCL 87) and HT-29 (BS TCL 132) cells were purchased from Istituto Zooprofilattico Sperimentale (Brescia, Italy). A co-culture was prepared by plating 40,000 cells/cm^2^ of a mixture of differentiated Caco-2 and HT-29 cells in a 70/30 ratio in complete RPMI 1640 medium [33]. The co-culture was grown in Transwell^®^ Millicell^®^ 24 insert plates (1.0 μm) assembled onto a Millicell^®^ 24-well receiver tray (Merck) and maintained in a complete RPMI-1640 medium for 10 d, i.e., 6 d after complete confluence, before the experiments [33]. To represent as best as possible the in vivo situation, where Ca absorption is also affected by the presence of other minerals such as bicarbonate and magnesium [32,34,35], a commercial Ca-rich water (11 mM Ca) was used. The in vitro digest of GP (DGP) and the Ca-rich water were separately administered to the apical side of the co-culture, mimicking the intestinal lumen. After metabolization, the content of the basolateral chamber was collected and submitted in vitro to the human osteoblast-like cells SaOS-2 (SaOS-2 HTB-85, ATCC, LGC standards, Sesto San Giovanni, Italy) [36] for assessing cell viability and proliferation. For determining the quantity of digest to be administered to the Caco-2 and HT-29 co-culture, a 50 g (DGP1x) or 100 g (DGP2x) daily assumption of GP was considered. The ratio µg of DGP/cm^2^ of the co-culture surface was calculated by considering that the volume of digest containing 50 g or 100 g of cheese would distribute in vivo over approximately 32 m^2^ of the small intestine surface. Accordingly, the administered water amount was calculated considering a two-liter daily assumption [20].

After 2 h, both the metabolized digests of GP (MDGP1x and MDGP2x) and the metabolized mineral water (MCa) contained in the basolateral chamber (800 μL) were collected. SaOS-2 cells, grown in 24-multiwell plates in Iscove’s Modified Dulbecco’s Medium (IMDM medium) at 70% of cell confluence, were treated with 100 μL of MDGP1x, MDGP2x or MCa in 1 mL (final volume) of IMDM medium. The amount of administered metabolized digests was calculated, considering both the growth surface of the SaOS-2 cells and the 1:2 dilution factor between the volume of the apical chamber (400 µL) vs. the basal one (800 µL). Treatments lasted 2 and 5 days. For 5 d observations, administration was repeated after the first 48 h. The effects of MDGP1x, MDGP2x and MCa on cell viability and proliferation were determined using an automatic cell counter EVE™ NanoEntek (VWR International, Milan, Italy), which provides the total cell count and the ratio of live cells/total cells. The results were reported as means from at least three independent experiments ± SD.

### 2.6. In Vivo Effects on Density and Volume of Bone in Mice Fed Diets Containing GP

Animal experiments were carried out in compliance with the institutional guidelines for the care and use of experimental animals (European Directive 2010/63/UE), approved by the Animal Use and Care Committee of the University of Milan and authorized by the Italian Ministry of Health (authorization number: 335/2020). Forty-eight male CD1 mice (3 weeks of age, Envigo, Huntingdon, UK) were kept in appropriate cages in an environment of 23 ± 1 °C and 50 ± 5% humidity, with a 12 h light/dark cycle and were fed ad libitum with a standard diet (Rat/Mouse Maintenance V1534 diet, sniff Spezialdiäten, Soest, Germany) for a week to permit their acclimatization. Subsequently, the mice were randomly divided into four groups (three mice per cage), each fed a specific custom-made isocaloric diet ad libitum (Table 1). These groups were as follows: Group 1: mice fed a diet of GP; group 2: mice fed a diet of CaCO_3_; group 3: mice fed a diet of low GP; group 4: mice fed a diet of high CaCO_3_. Grinded GP, used for the formulation of the diets, was partially defatted by warm centrifugation (16,000× *g*, 35 °C for 45 min) and the oily supernatant was subsequently removed. The gross composition (g/100 g) of the defatted GP was proteins 37.7, fat 16.6 and ash 5.1. The diet formulation and feed preparation were defined in accordance with a feed company (Mucedola srl, Settimo Milanese, Italy). The nutrients used were as follows: defatted GP, CaCO_3_, pea protein isolate, corn starch, maltodextrin, cellulose, sucrose and corn oil (Table 1). The diets that provided Ca from GP contained the defatted cheese, whereas it was not included in the diets where Ca was supplied by CaCO_3_. Corn oil was added to reach the same fat content (with the highest percentage in CaCO_3_-based diets). The added vitamins were as follows: A 6000 I.U./kg and D3 1500 I.U./kg. The minerals were as follows: phosphorus (monopotassium phosphate), potassium (monopotassium phosphate and potassium sulfate), sodium (sodium chloride) and magnesium (magnesium oxide). The trace elements (mg/kg) were as follows: Fe 40.2; Mn 10.5; Zn 33.5; Cu 6; I 6.0; Se 0.15; and Mo 0.15. The percentage of protein to kcal was 22.1%, the fat % kcal was 8.9% and the carbohydrate % kcal was 69%. The Gross Energy of all the diets was 18.7 MJ/kg (Table 1). The Ca supplied by the diet GP was 0.38% (*w*/*w*). As a control, the same amount of Ca was supplied by CaCO_3_ in the diet CaCO_3_. A lower amount of Ca (0.19%, *w*/*w*) provided by GP characterized the diet low GP (Table 1). Finally, the diet high with CaCO_3_ supplied a higher (0.68%, *w*/*w*) Ca content from CaCO_3_ (Table 1).

The food and water intake and the weight of the mice were monitored three times a week over the two months of experimentation.

Computed tomography (CT) and fluorescence acquisitions were performed by using the IVIS SPECTRUM/CT instrument (Perkin Elmer, Waltham, MA, USA). After 1 and 2 months, mice were anesthetized with a mixture of ketamine (100 mg/kg, Lobotor, Acme, Cavriago, Italy) and xylazine (8 mg/kg, Rompun, Elanco, Sesto Fiorentino, Italy) in PBS, and CT acquisition was performed. At the end of acquisition, the 3D Regions of Interest (ROI) were selected on the femur, tibiae, knee and tail vertebrae (Supplementary Figure S1), and then the regions were measured as absorption coefficient (total value/number of 3D ROI voxel) or Hounsfield unit (total Hounsfield value/number of 3D ROI voxel).

At the same time points of CT acquisition, the Osteosense 800 fluorescent probe, an in vivo fluorescent diphosphonate imaging agent (Perkin Elmer, Waltham, MA, USA), was intravenously administered to 4 mice from each group that were randomly chosen (2 nmol/25 g mouse weight); after 24 h (recommended optimal biodistribution time), the mice were anesthetized (as previously described) and fluorescence imaging was performed. Finally, all the images were scaled with the same color scale and quantified by applying a two-dimensional ROI to the mice’s bodies using the Living Image 4.7.4 Software (Perkin Elmer). Data were presented as average radiance efficiency (photons/seconds/cm^2^/steradian)/(µW/cm^2^), which is a normalized measure of emitted photons that considers all acquisition parameters.

At the end of the experiment, the animals were sacrificed by cervical dislocation after anesthesia, as previously described. The bones of the hind legs were separated from the muscle tissue, and they were subjected to CT in a clinical scan (GE LightSpeed 16 scanner, GE Healthcare, Chicago, IL, USA). The X-ray generator was activated with a voltage of 120 kVp, a current of 40 mA and a scan time of 2 s. Tomographic images were reconstructed using an iterative method with 0.48 mm-thick sections. The images obtained in DICOM format were analyzed with the open-source 3D Slicer 5.6.2 software [37], a software for the visualization and analysis of biomedical images. The bone density and volume of each femur were obtained by automatic segmentation, accepting density values greater than 80 Hounsfield units (to exclude any tissue that may have been adhered to the femurs). Mean and maximum density values were analyzed.

### 2.7. Statistical Analysis

The in vitro data were statistically analyzed using a three-way ANOVA, both on data from the single experiments and on data grouped as reported on the graph using Prism 4 software (GraphPad Software Inc., San Diego, CA, USA).

The data obtained ex vivo were analyzed using the SAS 9.4 software (SAS Inst., Cary, NC, USA) while adopting a randomized block as an experimental model, where the block was the day of incubation and the single Ussing chamber was the experimental unit.

For in vivo experiments, the number of mice per group was defined by a priori analysis with G*power 3.1 software [38,39], using the following parameters: Test F, ANOVA: fixed effects, omnibus, one-way; effect size: 0.5; α error = 0.05; power = 0.8; comparison among groups (4 different diets). Each group was composed of 12 mice (no animals or data were excluded by results). For the in vivo experiments, the randomized block approach was used, where the blocks were the different days of feeding diet, and the experimental unit was the cage. Imaging data were analyzed by using a *t*-test.

## 3. Results and Discussion

### 3.1. Characterization of the CPP Profile of GP Digest

We assessed the qualitative CPP profile of the GP sample after in vitro SGID, using the internationally harmonized protocol proposed by the INFOGEST network. UPLC/HR-MS revealed 161 unique CPP in the digest, and they included mono-, di-, and tri-phosphorylated peptides deriving from α_S1_-, α_S2_-, β-, and κ-CN (Table 2). The length of CPP ranged from 6 to 24 amino acids, and among them 26 were tri-phosphorylated and 15 contained the sssEE cluster responsible for Ca-binding properties [40,41]. Tri-phosphorylated CPP containing this cluster mainly represented fragments released from the sequences f(59–74) of α_S1_-CN, f(6–15) and f(53–61) of α_S2_-CN and f(15–24) of β-CN (Table 2). Six of these CPP were derived from β-CN and these are the most effective peptides for mineral binding activity, as reported in the literature [42,43].

### 3.2. Ex Vivo Evaluation of Ca Absorption

We used the GP digest for the ex vivo experiment, applying it on Ussing chambers mounted with segments of rat ileum tissue sourced from adult animals. The intestinal absorption of Ca can be active or passive (diffusion driven by the difference in Ca concentration between the inside and outside) depending on the concentration of Ca in the intestinal lumen. This feature made it difficult to compare different levels of Ca ingestion and, therefore, the intestinal concentration of this mineral.

For this reason, we decided to study intestinal absorption only in the ileum, an intestinal segment where Ca is absorbed in passive way (paracellular). This means that it occurs through the tight junctions among the enterocytes. Therefore, absorption is not affected by the concentration of the mineral [44]. The obtained results showed that Ca absorption was significantly (*p <* 0.0001) higher using GP digest compared to that observed for CaCO_3_ solution (1.35 mM) (Table 3).

Nonetheless, Ca absorption measured ex vivo for GP digest was lower than that (83.6%) reported in vivo for rats [24]. Conversely, the obtained data were within the values (23.6–47.5%) revealed by previous studies regarding Ca absorption using aqueous solution [45,46,47]. These studies found the bioavailability of Ca-rich water (300–400 mg/L) to be slightly higher (8.4 ± 4.3%) than that of milk. Nonetheless, they were conducted on humans, but with low Ca intake (10–25% of the adult allowance). Under these conditions, the body absorbs Ca through an active transport system, thus masking the role of paracellular transport responsible for Ca absorption from dairy products, i.e., in the presence of higher Ca intake.

From this point of view, the equal Ca absorbability from mineral water and milk or dairy products reported by previous experiments on humans [34] could depend on individual physiological factors that are difficult to consider due to their day-by-day variability and due to their different experimental designs (serum and urine analyses, various categories of subjects). In our ex vivo model, the active Ca transport system was not present, and this also explains the differences with the data obtained in vivo, where Ca could be absorbed throughout the small intestine. The greater intestinal absorption of the Ca contained in the GP compared to that dissolved in CaCO_3_ may be related to the presence of CPP in GP digest. Due to their negative charge, CPP bind Ca ions, thus forming aggregates which maintain the mineral in a soluble form and allow the interaction with intestinal cells [44]. Moreover, CPP accumulate at the distal intestinal tract, where the absorption of Ca is paracellular.

### 3.3. In Vitro Effects of a GP Digest on Viability and Proliferation of Osteoblast-like Cells

The intestinal absorption of nutrients is affected by the absorptive surface, which is constituted by different cell phenotypes. Among these, the absorptive and the goblet cells contribute to nutrient absorption through the intestinal barrier and the mucus layer [48]. On this basis, we administered the digest of GP and the Ca-rich water (11 mM Ca) as a control to the apical side of a Caco-2/HT-29 cell 70/30 co-culture, using the model of a human intestinal epithelium. This co-culture represents a cell model of absorptive (Caco-2) and mucus-secreting (HT-29) cells. Indeed, this model is more reliable for simulating nutrient absorption in comparison to a Caco-2 cell monolayer [32,48]. Two different amounts of GP digest were administered to mimic an intake of 50 g (DGP1x) or 100g (DGP2x) of cheese. The former amount is the daily assumption recommended by the Italian Dietary Guidelines. It refers to a standard diet of 2000 kcal with a frequency of consumption equal to three cheese portions a week [49]. The 100 g intake was used to consider a potential dose-dependent effect.

**Figure 1:**
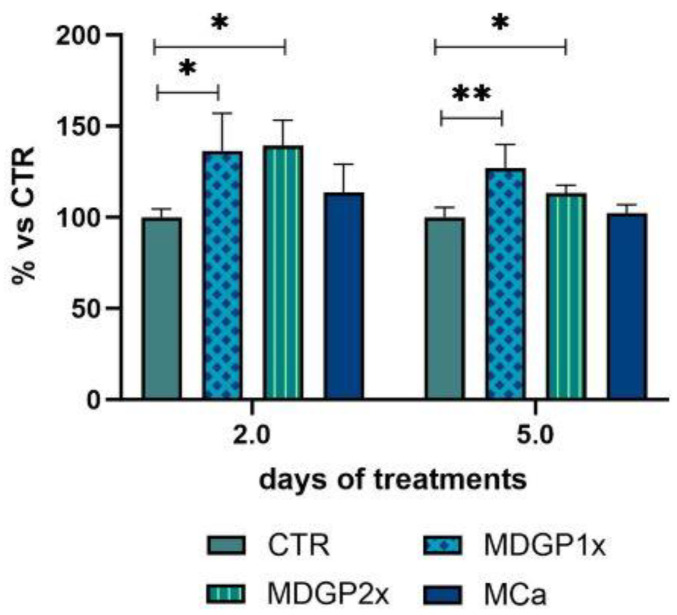
Effects of the metabolized digests of Grana Padano (MDGP1x and MDGP2x) and metabolized Ca-rich drinking water (MCa) on SaOS-2 cell proliferation in vitro. Bars represent the mean ± standard deviation from three independent experiments. * *p <* 0.05 vs. CTR; ** *p <* 0.01 vs. CTR. (CTR, control).

This experimental approach was meant to simulate the in vivo absorption through the intestinal lumen [32]. Thus, after 2 h treatment, we recovered, from the basolateral chamber of the Caco-2/HT-29 co-culture, the two metabolized digests of GP (MDGP1x and MDGP2x) and Ca-rich water (MCa). Subsequently, we administered them to SaOS-2 cells as an in vitro model of human osteoblast-like cells. SaOS-2 cell viability was not affected upon administration and the live cells ranged between 95.55 ± 1.97% and 87.08 ± 9.89% of total cells after 2 or 5 days. The administration of MDGP1x and MDGP2x resulted in an increase in the number of SaOS-2 cells after 2 d incubation (+36% and +39% vs. control cells, respectively, *p <* 0.05). The recorded proliferative effect persisted after 5 d treatment (+27% and +13% vs. control) for both MDGP1x (*p <* 0.01) and MDGP2x (*p <* 0.05) (Figure 1). On the contrary, incubation with MCa never resulted in a significantly different proliferation of SaOS-2 cells in comparison to the control (Figure 1). Overall, the in vitro experiment highlighted that MDGP1x or 2x activated SaOS-2 proliferation, thus showing a potential positive effect on bone cells [47]. This finding likely relates to the cheese matrix effect, as demonstrated both in vivo (postmenopausal women) and in vitro (using SaOS-2 cells) for dairy products [50].

The increased osteoblast proliferation is the result of the GP matrix which contains bone growth factors such as Ca ions and CPP, as well as other peptides. Apart from the mineralization role, the increase in the extracellular Ca induces the intracellular Ca transients. They act as a second messenger, able to activate some specific signaling pathways including the proliferation, differentiation and apoptosis of osteoblasts, thus directly affecting bone remodeling [51]. Due to their ability to bind and solubilize Ca ions, commercial CPP mixtures, as well as purified sequences, have been demonstrated to positively affect osteoblast proliferation and the expression and activity of alkaline phosphatase. This enzyme is a marker of human osteoblast differentiation through the stimulation of Ca uptake by the L-type Ca channels [36].

The bone health effects revealed using osteoblast cells in vitro could also be due to the action of peptides other than CPP. Different peptides released from CN fractions have been found to show osteoanabolic activity through the enhancement of differentiation in primary rat osteoblast cells [8]. Other peptides found from milk fermented by *Lactobacillus helveticus* were investigated for their effects on the proliferation, differentiation and mineralization of MC3T3-E1 cells, an osteoblast precursor cell line derived from mouse [52]. The underlying mechanisms involve diverse biochemical phenomena, including the activation of the extracellular signal-regulated kinases (ERK1/2) and Protein kinase B (AKT) pathways, known to promote the expression of osteogenic genes such as RUNX2 and ALP. These genes enhance the proliferative activity and differentiation of osteoblasts [52]. Finally, the activation of mitogen-activated protein kinase (MAPK) takes place, thus resulting in the activation of the proliferation, differentiation and extracellular matrix formation in osteoblasts [52]. Overall, CPP and osteoblast differentiation-enhancing peptides would likely support the healthy bone properties revealed in the in vitro experiments using SaOS-2 cells [53]. Nonetheless, whether osteogenic peptides are absorbed in the gastrointestinal tract remains unclear, and so far, there is scarce evidence for the effect of milk osteogenic peptides on bone formation in humans [8].

### 3.4. In Vivo Effects on Density and Volume of Bone in Mice Fed Diets Containing GP

Considering the findings from ex vivo and in vitro experiments, we assessed the effectiveness of GP in increasing bone density and bone remodeling in vivo. To this aim, we fed the CD1 mice four different diets containing GP or CaCO_3_ as a Ca source (Table 1). To maintain the fat content of the diets the same as that in a standard maintenance one for rodents, we partially defatted GP by warm centrifugation without using organic solvents to avoid potential toxic effects on the animals.

We measured food intake and mouse weight three times a week during experimentation. They fluctuated among the mice groups fed different diets (Supplementary Figure S2 and Figure S3, respectively). The differences were statistically significant for food consumption among the different diets at the first time points, likely due to the adaptation of the animals to the diet. For water intake, we observed a statistically significant difference only in the middle of the experimentation, and it was not constant over time (Supplementary Figure S2). This finding is probably related to variability among cages, rather than being caused by the diets themselves. This difference did not reflect biological variance; indeed, variability in food and water intake did not reflect the different growth among the animal groups over time (Supplementary Figure S3). Nonetheless, we detected some variability among individuals (Supplementary Figure S3 and Table 4).

**Figure 2:**
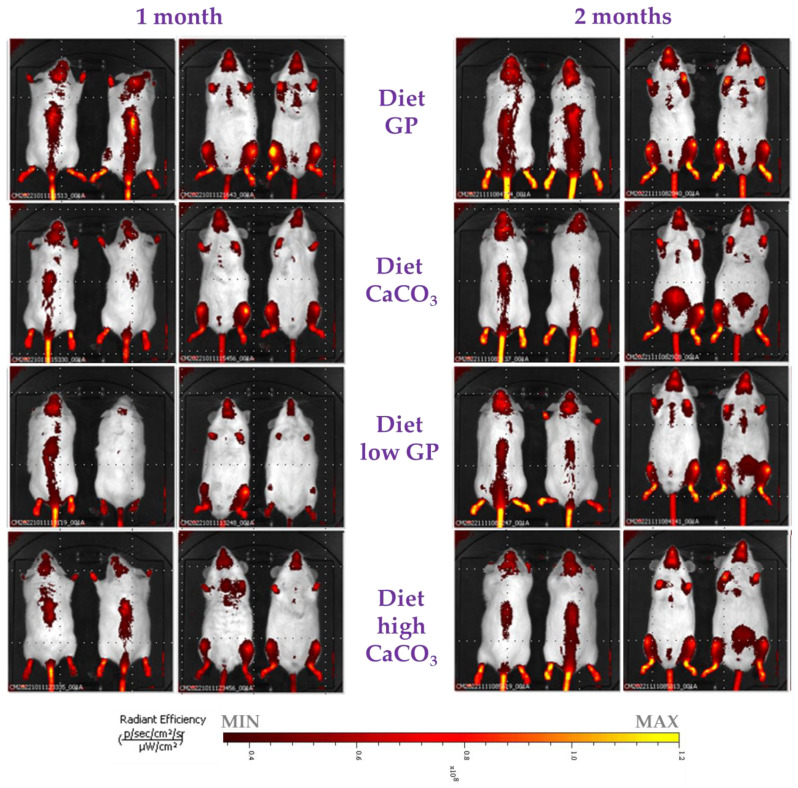
Images of Osteosense fluorescent signal in mice after 1 and 2 months of feeding different diets (dorsal and supine view). The same color scale was applied to the images.

**Figure 3:**
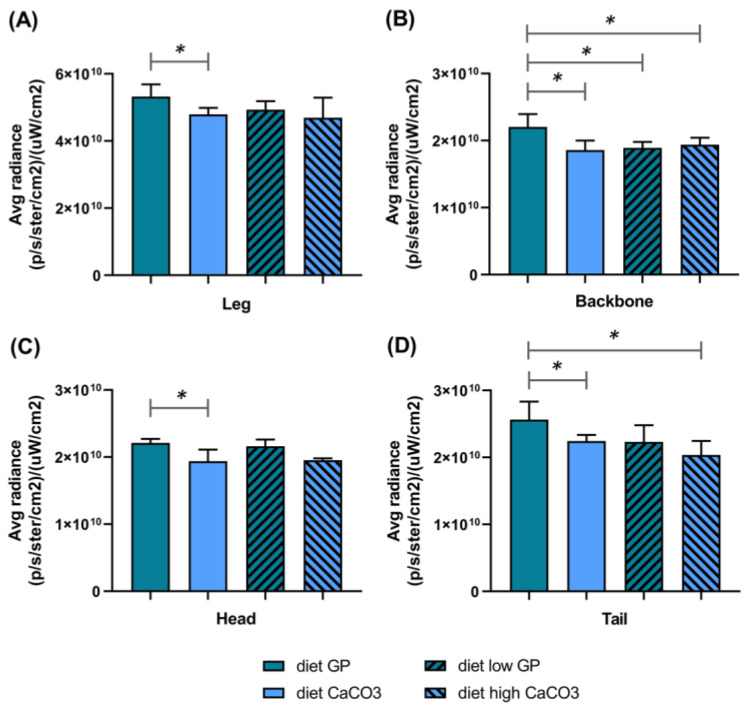
The in vivo determination of bone remodeling in different mice body districts by the quantification of a fluorescent signal (Osteosense 800 probe) after a 2-month feeding program of four different diets. The bars represent the mean ± standard deviation of signals in the different body districts of mice ((**A**) leg; (**B**) backbone; (**C**) head; (**D**) tail) (* *p <* 0.05). (Avg, average).

We evaluated the bone activity in different body districts using a fluorescent probe (Osteosense) that was able to quantitatively monitor bone growth and resorption [54,55,56,57,58] (Figure 2 and Figure 3). This approach allowed us to monitor bone remodeling in vivo through a non-invasive method. To the best of our knowledge, this is the first time a fluorescent probe has been used to monitor the effect of different diets in mice. Indeed, to date, it is mainly used to evaluate the extent of bone reformation after bone injury in both traumatic and tumoral lesions [55,58]. In our study, we employed this probe to assess whether the Ca supplied by different sources (GP or CaCO_3_) could induce differences in terms of bone remodeling.

After one month, a more intense signal was observed at leg level in animals fed a diet of GP than in those fed with other diets. Indeed, the values ([p/s/ster/cm^2^]/[µW/cm^2^]) as a sum of the four legs were as follows: diet GP = 1.95 × 10^11^, diet CaCO_3_ = 1.79 × 10^11^, diet low GP = 1.74 × 10^11^ and diet high CaCO_3_ = 1.78 × 10^11^ (*t*-test diet GP vs. diet CaCO_3_ *p* = 0.02). After two months, we detected a significantly higher signal the in posterior legs of mice fed a diet of GP in comparison to mice fed a diet of CaCO_3_ (Figure 3A). Moreover, we observed a statistically significant difference in the quantified fluorescent signal between diet GP and diet CaCO_3_ as the controls in other different body districts (Figure 3). In these districts, such as the backbone, tail and head, we measured a higher signal in mice fed a diet of GP compared to animals fed a diet of CaCO_3_. At the head level, we observed a similar fluorescent signal between mice fed diets of GP and low GP. Interestingly, when mice were fed a diet of high CaCO_3_, even supplying double the amount of Ca provided by CaCO_3_, the fluorescent signal monitored by using Osteosense was not improved (Figure 3). Overall, the signal in mice fed a diet of GP was generally higher than that observed for their counterparts fed diets of CaCO_3_ and high CaCO_3_.

**Figure 4:**
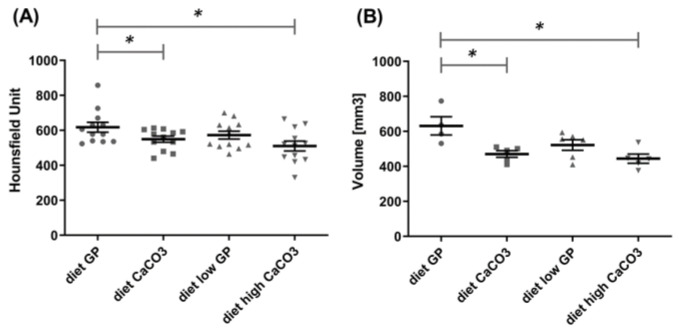
The computed tomography quantification of mice bone density (as Hounsfield units) at the level of tail vertebra (**A**) and bone volume of posterior legs (expressed as mm^3^) (**B**). The bars represent the mean ± standard deviation of bone density or volume (* *p <* 0.05).

Computed tomography represents the imaging technique most used to monitor bone density. At the end of the experiment, we performed the CT acquisition to evaluate the radiodensity of bones, using a translational approach. Indeed, X-rays are used in the clinics to evaluate bone density and mineralization [59]. This is true for humans but also for mice [60,61]. The density was quantified in Hounsfield units by applying 3D ROIs on the femur, tibiae, knee and tail vertebrae to reproduce the internationally accepted measurement of human bone mineral density by dual-energy X-ray absorptiometry, evaluating the spine to discriminate between healthy bones, osteopenia and osteoporosis [62]. As for the fluorescent signal, we observed significantly different data in the tail and vertebrae between mice fed a diet of GP and CaCO_3_. Contrarily, no differences were recorded between mice fed a diet of GP and low GP. Mice fed with a diet (high CaCO_3_) providing double the Ca amount from CaCO_3_ did not see any improvement in bone density (Figure 4A).

After sacrifice, the bones of the animals’ hind legs were collected, and their muscles were removed and subsequently acquired with a clinical CT, exploiting the higher energy of the X-rays associated with this instrument. The analysis showed a greater bone volume in the mice group fed a diet of GP compared to a control diet of CaCO_3_. This difference was statistically significant (Figure 4B).

Overall, animals fed diet a of GP showed increased bone mineralization, volume and augmented radiodensity compared to those fed control diets in which Ca was provided as CaCO_3_.

The described findings were obtained in a murine model ranging from young to adult age, since the experimentation started at 3 weeks of age (considered young mice) and lasted until 3 months of life (deemed adult). Moreover, we used healthy mice to observe differences without confounding variables due to the presence of diseases (such as osteoporosis) or genetic alterations (e.g., TghuRANKL or B6-hRANKL transgenic models). Based on this, it can be speculated that the cheese matrix as a whole is responsible for better bone health in mice fed a diet with GP.

## 4. Conclusions

The results obtained ex vivo demonstrated the paracellular intestinal absorption of Ca when digested GP was put in contact with the ileum. Despite the measured Ca absorption for GP digest being lower than that reported in vivo for rats, it significantly exceeded that observed for CaCO_3_ solution. Using in vitro cell cultures, we demonstrated the increase in osteoblast differentiation and the proliferation of GP digests. On the contrary, this positive effect was not revealed when we administered the Caco-2/HT-29 co-culture metabolized mineral water to SaOS-2 cells. Outcomes from fluorescent probe and CT highlighted in vivo higher bone matrix apposition and bone remodeling in mice fed GP compared to the other studied diets, which did not contain this cheese as a protein and Ca source. Nonetheless, despite the less effective outcomes obtained with Ca-rich water and CaCO_3_ solution, mineralized water could potentially contribute to reaching the required daily amounts of Ca and the desired level of bone health.

Overall, these results highlight the role of the GP matrix in supporting bone health effects, and they enforce the function of GP as an important component for improving Ca uptake and ameliorating bone mass as part of a balanced diet. Using these findings as a basis, further studies are necessary to deeply understand the long-term effect of the Ca provided by GP in humans. Moreover, additional research is needed to strengthen the importance of GP intake in improving bone remodeling and density, especially in aging and pathological conditions such as osteoporosis.

## Figures and Tables

**Table 1:** Composition (g/100 g) of the diets used for mice feeding.

	Diet			
	GP	CaCO_3_	Low GP	High CaCO_3_
Grana Padano	27.00	0.00	13.50	0.00
CaCO_3_	0.00	0.85	0.00	1.70
Pea protein isolate	0.00	20.00	10.00	20.00
Corn starch	39.00	37.00	39.00	37.00
Maltodextrin	13.20	13.20	13.20	13.20
Cellulose	5.00	5.00	5.00	5.00
Sucrose	10.00	10.00	10.00	10.00
Corn oil	0.00	8.00	3.50	8.00
Vitamins	1.00	1.00	1.00	1.00
Minerals and trace elements	3.50	3.50	3.50	3.50
Choline	0.25	0.25	0.25	0.25
L-Cysteine	0.30	0.30	0.30	0.30
Gross Energy (MJ/kg)	18.70			

**Table 2:** Caseinophosphopeptides (CPP) identified in Grana Padano cheese digest: number, parent casein (CN) and phosphorylation degree (1P, 2P and 3P represent mono-, bi- and tri-phosphorylated CPP, respectively). The sequence of tri-phosphorylated CPP containing the sssEE amino acid motif is also reported.

α_S1_-CN	α_S2_-CN	β-CN	κ-CN
1P–37	1P–33	1P–24	1P–1
2P–23	2P–12	2P–5	2P–0
3P–10	3P–10	3P–6	3P–0
(sssEE–4)	(sssEE–5)	(sssEE–6)	(sssEE–0)
f(59–74) (K)-QMEAESIsssEEIVPN-(S) f(64–74) (E)-ISIsssEEIVPN-(S) f(65–74) (S)-IsssEEIVPN-(S) f(66–74) (I)-sssEEIVPN-(S)	f(6–15) (E)-H*Vs*ssEESII-(E) f(7–14) (H)-*Vs*ssEESI-(I) f(8–14) (V)-sssEESI-(I) f(53–61) (Y)-SIGsssEES-(I) f(56–61) (G)-sssEES-(I)	f(15–23) (E)-SLsssEESI-(T) f(15–24) (E)-SLsssEESIT-(R) f(16–23) (S)-LsssEESI-(T) f(16–24) (S)-LsssEESIT-(R) f(17–23) (L)-sssEESI-(T) f(17–24) (L)-sssEESIT-(R)	

**Table 3:** Absorption of calcium present in Grana Padano digest and CaCO_3_ solution during ex vivo experiments with Ussing chambers.

	Ca Absorbed (%)	Standard Error	*p*-Value
Grana Padano digest	74.84	3.23	<0.0001
CaCO_3_ solution	44.27	3.23	

**Table 4:** Statistical analysis of initial and final mice weight, food intake and mice weight increase. RMSE: mean square error; *p*: *p*-value.

Diet	Total Weight Gain (8 Weeks), g	Total Feed Intake (8 Weeks), g	Feed Weight Gain Ratio
GP	15.31	119.71	7.86
CaCO_3_	16.77	109.46	6.84
low GP	16.64	110.53	6.67
high CaCO_3_	14.90	108.64	7.47
RMSE	2.82	8.31	1.16
GP vs. CaCO_3_	0.55	0.11	0.28
CaCO_3_ vs. high CaCO_3_	0.50	0.89	0.58
GP vs. low GP	0.25	0.15	0.03

## Data Availability

The original contributions presented in this study are included in the article/Supplementary Material. Further inquiries can be directed to the corresponding author.

## Supplementary Materials

The following supporting information can be downloaded at https://www.mdpi.com/article/10.3390/foods14020273/s1: Figure S1. Representative image of 3D Regions of Interest (ROI, in red) positioned on different bones of a reference mouse; Figure S2. Monitoring of water and food intake in mice over the experimental period; Figure S3. Mice weight over the experimental period.
