# Supplementary material for: Ex Vivo, In Vitro and In Vivo Bone Health Properties of Grana Padano Cheese
Source: Foods. 2025 Jan 16;14(2):273. doi: 10.3390/foods14020273 (PMC11765351; doi:10.3390/foods14020273)

<u>Supplementary material</u>

Figure S1. Representative image of 3D Regions Of Interest (ROI, in red) positioned on different bones of a reference mouse.

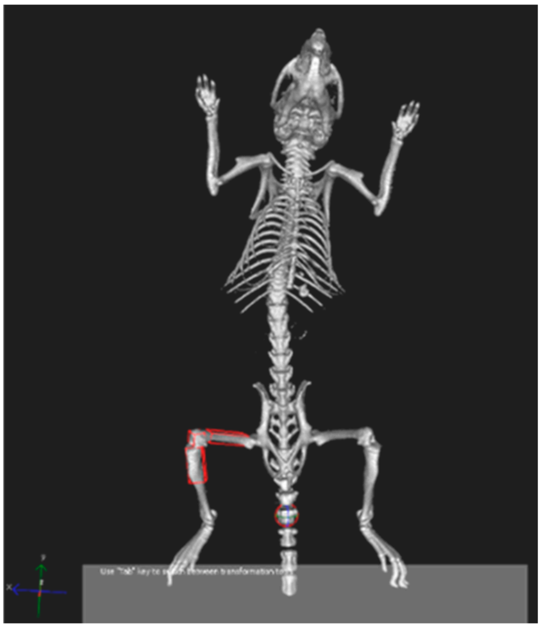

Figure S2. Monitoring of water and food intake in mice over the experimental period.

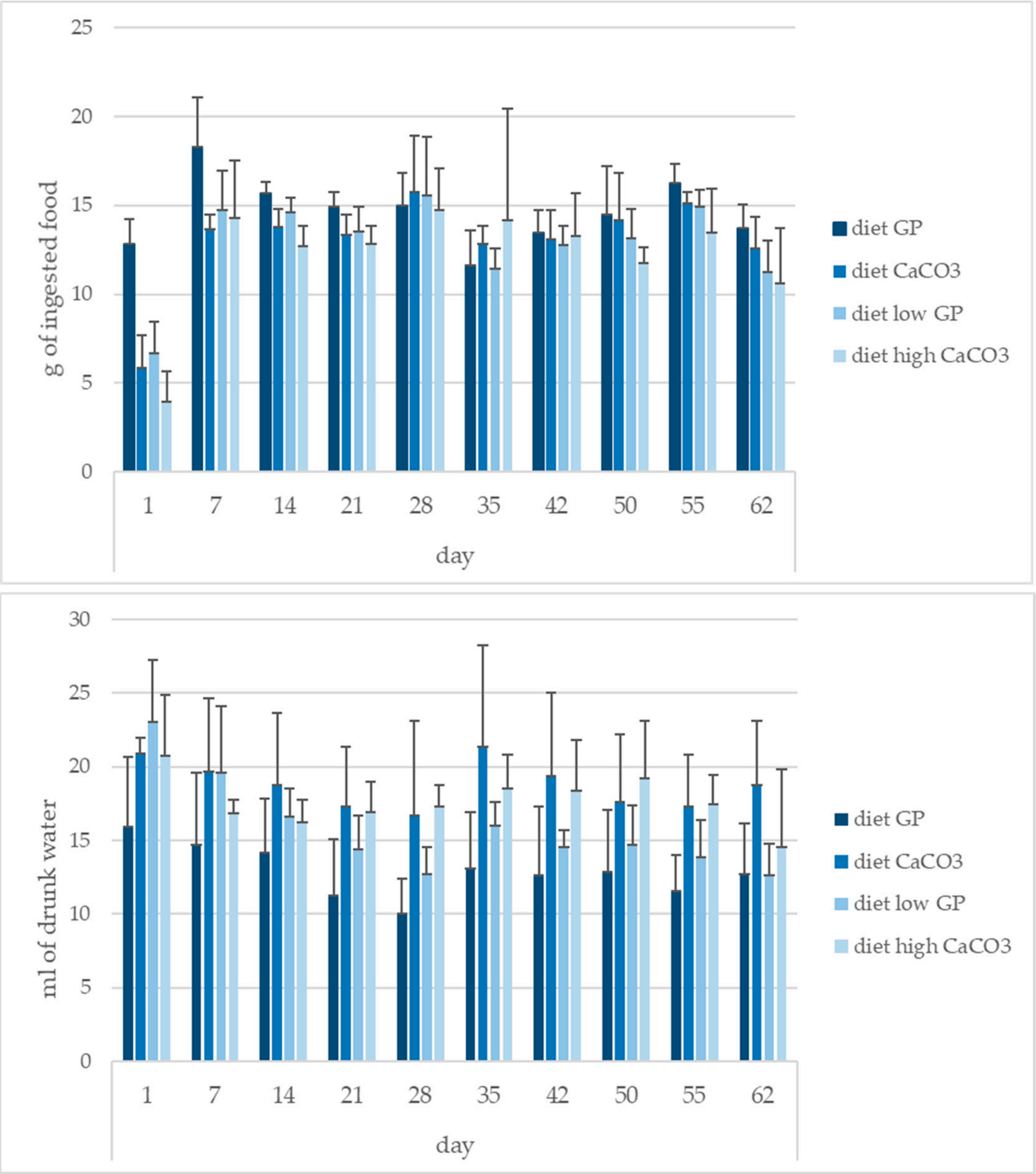

Figure S3. Mice weight over the experimental period.

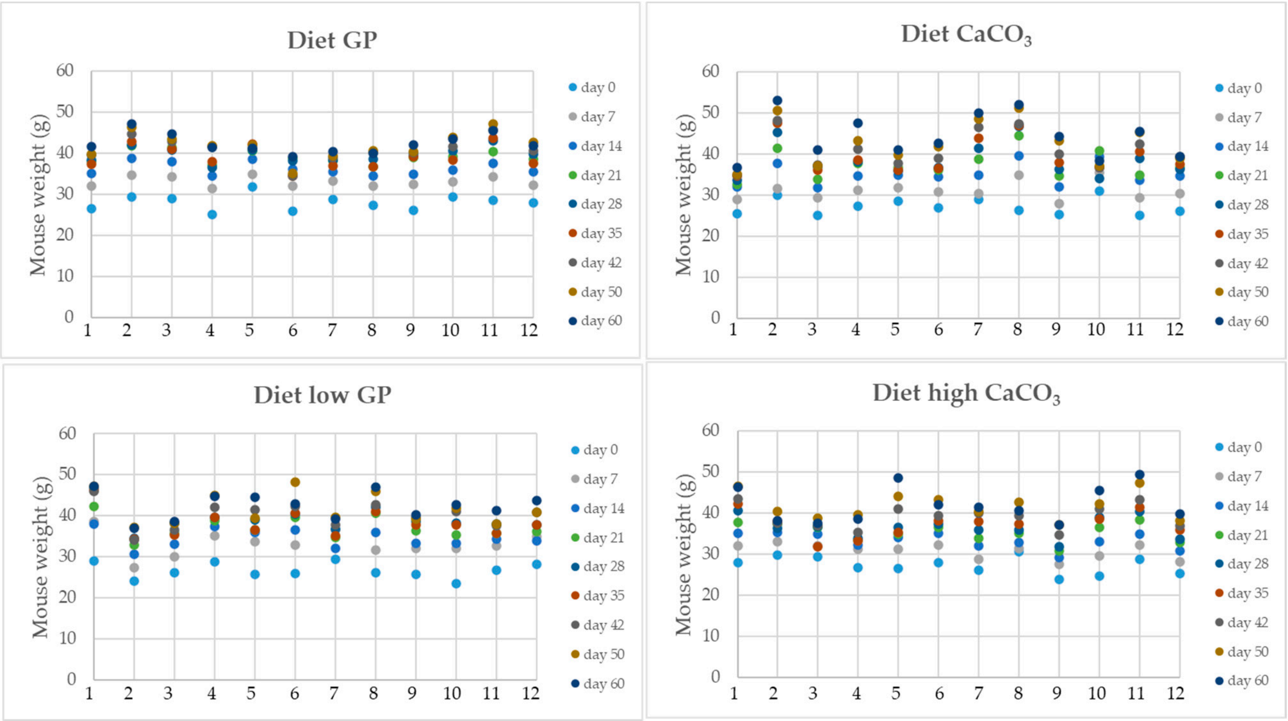

Supplement: Supplementary file 1 [file foods-14-00273-s001.zip › foods-3387825-supplementary.pdf]
